# Development and validation of a nomogram for predicting all-cause mortality in American adult hypertensive populations

**DOI:** 10.3389/fphar.2023.1266870

**Published:** 2023-11-22

**Authors:** Long Yang, Xia Shen, Zulihuma Seyiti, Jing Tang, Abudushalamu Kasimujiang, Tuohutasheng Dejite, Ling Zhao, Xue-Feng Shan, Xiao-Ming Gao

**Affiliations:** ^1^ State Key Laboratory of Pathogenesis, Prevention and Treatment of High Incidence Diseases in Central Asia, College of Pediatrics, Xinjiang Medical University, Clinical Medical Research Institute of Xinjiang Medical University, Urumqi, China; ^2^ Department of Nursing, Wuxi Medical College of Jiangnan University, Wuxi, China; ^3^ Clinical Laboratory, First Affiliated Hospital of Xinjiang Medical University, Urumqi, China; ^4^ Xinjiang Key Laboratory of Medical Animal Model Research, Urumqi, China; ^5^ Department of Cardiology of the First Affiliated Hospital of Xinjiang Medical University, Urumqi, China; ^6^ Pediatric Cardiothoracic Surgery, First Affiliated Hospital of Xinjiang Medical University, Urumqi, China

**Keywords:** nomogram, hypertension, NHANES, LASSO, mortality, machine learning

## Abstract

**Backgrounds:** Hypertension stands as the predominant global cause of mortality. A notable deficiency exists in terms of predictive models for mortality among individuals with hypertension. We aim to devise an effective nomogram model that possesses the capability to forecast all-cause mortality within hypertensive populations.

**Methods:** The data for this study were drawn from nine successive cycles of the National Health and Nutrition Examination Survey (NHANES) spanning the years from 1999 to 2016. The dataset was partitioned into training and validation sets at a 7:3 ratio. We opted for clinical practice-relevant indicators, applied the least absolute shrinkage and selection operator (LASSO) regression to identify the most pertinent variables, and subsequently built a nomogram model. We also employed concordance index, receiver operating characteristic (ROC) curves, calibration curves and decision curve analysis (DCA) to assess the model’s validity.

**Results:** A total of 17,125 hypertensive participants were included in this study with a division into a training set (11,993 individuals) and a validation set (5,132 individuals). LASSO regression was applied for the training set to obtain nine variables including age, monocytes, neutrophils, serum albumin, serum potassium, cardiovascular disease, diabetes, serum creatinine and glycated hemoglobin (HbA1C), and constructed a nomogram prediction model. To validate this model, data from the training and validation sets were used for validation separately. The concordance index of the nomogram model was 0.800 (95% CI, 0.792–0.808, *p* < 0.001) based on the training set and 0.793 (95% CI, 0.781–0.805, *p* < 0.001) based on the validation set. The ROC curves, calibration curves, and DCA curves all showed good predictive performance.

**Conclusion:** We have developed a nomogram that effectively forecasts the risk of all-cause mortality among American adults in hypertensive populations. Clinicians may use this nomogram to assess patient’s prognosis and choose a proper intervention in a timely manner.

## Introduction

Hypertension stands as the most prevalent chronic ailment globally, which afflicts nearly 40% of adults across the world, and its prevalence continues to rise each year according to World Health Organization report (Pradhan et al., 2020). Prolonged elevation in blood pressure initiates a cascade of physiological changes, including vascular endothelial damage, abnormal proliferation, migration, invasion and phenotypic transformation of vascular smooth muscle cells (VSMCs). These processes eventually cause endothelial dysfunction, blood vessel constriction and stiffness, ultimately, insufficient blood supply to crucial organs like the heart, kidney and brain (Fang et al., 2019; Dikalova et al., 2020; Huang et al., 2020). Consequently, hypertension becomes a significant risk factor for the development of cardio- and cerebro-vascular diseases. In the United States alone, cardiovascular disease claims over 600,000 lives annually, with hypertension either directly responsible for or contributing significantly to more than 400,000 of these fatalities (Saltzgiver et al., 2019). Understanding the pathogenesis and predicting prognosis is essential to making informed decisions about the management of hypertension (Agongo et al., 2020). Early identification of high-risk patients with hypertension can improve their life expectancy and reduce the prevalence of life-threatening complications (Ma et al., 2020).

Machine learning represents a branch of artificial intelligence that possesses the capability to autonomously discern patterns within sample data and thereby enhance the accuracy of predictions (Chen et al., 2020). Noteworthy machine learning techniques encompass random forests, neural network, and the least absolute shrinkage and selection operator (LASSO) regression (Bayer et al., 2020). In recent years, various machine learning methods have been widely used for prognosis prediction of clinical patients, including cancer, cardiovascular diseases, and after surgical interventions. (Chang et al., 2013; Wu X. et al., 2022; Wang et al., 2022). Researchers have substantiated that machine learning techniques can provide more accurate diagnoses or prognoses compared to conventional statistical approaches (Wang et al., 2021).

Currently, many studies from large populations have observed that several metrics are associated with long-term prognosis in people with hypertension. For example, body mass index (BMI), white blood cells, uric acid (UA), and anti-hypertensive drugs (Zhu J. et al., 2022; Buonacera et al., 2022; Lu et al., 2022; Yan et al., 2022). Nevertheless, most of these studies have primarily focused on evaluating the prognostic impact of individual risk factors. There remains a notable scarcity of predictive models that incorporate multiple variables (comprising several risk factors) to ascertain the risk of mortality within hypertensive populations. In many cases, nomograms have proven to be a valuable tool for precisely estimating the risk of disease or complications (Wang et al., 2013). In the current study, we have harnessed data sourced from the National Health and Nutrition Examination Survey (NHANES) to undertake the development and validation of a straightforward, practical, and accurate predictive tool. This tool aims to effectively identify the risk of all-cause mortality among individuals with hypertension.

## Methods

### Study design and participant selection

The data were extracted from nine consecutive cycles of the National Health and Nutrition Examination Survey (NHANES), spanning from 1999 to 2016. The NHANES database uses a complex sampling design and is mobilized by the National Center for Health Statistics of the United States Centers for Disease Control and Prevention (Zipf et al., 2013). Mobile Examination Centers (MECs) were utilized to administer physical examinations and collect blood samples from study participants, with these tasks being performed by extensively trained personnel (Ruan et al., 2022). The data were analyzed from February 2023 to March 2023. This study strictly followed Transparent Reporting of a multivariable prediction model for Individual Prognosis or Diagnosis (TRIPOD) (Collins et al., 2015). Furthermore, ethical approval for this study was granted by the National Center for Health Statistics (NCHS) Ethics Review Board, under the protocols #98–12, continuation of #2005–06, #2011–17, and continuation of #2011–17. Detailed information regarding these ethical approvals can be accessed via the following website: https://www.cdc.gov/nchs/nhanes/irba98.htm. All data used in this study are publicly available on the following website: https://www.cdc.gov/nchs/nhanes. Additionally, each participant provided written informed consent prior to their inclusion in the study.

### Diagnostic criteria for hypertension

Hypertension was defined as meeting one or more of the following criteria (Chobanian et al., 2003; Wu LD. et al., 2022): 1. Self-reported history of hypertension. 2. Use of antihypertensive medication. 3. Systolic blood pressure (SBP) ≥140 mmHg. 4. Diastolic blood pressure (DBP) ≥90 mmHg. BP were measured by a trained physician, employing a mercury sphygmomanometer equipped with an appropriately sized cuff. To ensure accuracy, three separate blood pressure measurements were taken for each participant, and the average of these three readings was used for analysis.

### Predictive variable selection

The selection of predictive variables for this analysis was based on a combination of clinical expertise and prior findings in the existing literature (Oparil et al., 2018). First, essential demographic data such as age, gender, race, marital status, education level, height, weight, and BP were gathered. Second, A wide array of biological markers and measurements were included in the dataset. These encompassed counts of white blood cells, red blood cells, lymphocytes, neutrophils, monocytes, and platelets, as well as measurements of hemoglobin, serum uric acid, serum creatinine, high density lipoprotein cholesterol (HDL-C), low density lipoprotein cholesterol (LDL-C), triglycerides, albumin, sodium, potassium, and glycated hemoglobin (HbA1C). Information regarding specific medical conditions and medication use was collected through structured questionnaires. This included data related to cardiovascular disease (CVD), diabetes mellitus, and the use of antihypertensive medications. Race was categorized as non-Hispanic white, non-Hispanic black, Mexican American, or others. Antihypertensive drugs were classified into categories such as angiotensin-converting enzyme inhibitors/angiotensin receptor blockers (ACEI/ARB), calcium channel blockers, beta-blockers, and diuretics. Statins were defined as drugs used to manage hyperlipidemia. CVD data were gathered from self-reported information obtained during personal interviews. Participants were asked whether they had ever been diagnosed by a healthcare professional with conditions such as congestive heart failure (CHF), coronary heart disease (CHD), angina, heart attack, or stroke. If any of these questions were answered affirmatively, the presence of CVD was considered. Diabetes mellitus (DM) was identified through multiple criteria, including self-report of a doctor’s diagnosis, glycohemoglobin (HbA1c) levels exceeding 6.5%, fasting glucose levels equal to or greater than 7.0 mmol/L, random blood glucose levels equal to or greater than 11.1 mmol/L, 2-h oral glucose tolerance test (OGTT) blood glucose levels equal to or greater than 11.1 mmol/L, or the use of diabetes medications or insulin.

### Follow-up results

The assessment of the mortality status of the study population was conducted using the NHANES Public-Use Linked Mortality Files, with data up to the termination date of 31 December 2019. This linkage was established through a probability matching algorithm, connecting the National Center for Health Statistics (NCHS) database with the National Death Index (NDI) (Zhou et al., 2021). In this context, all-cause mortality was defined as death resulting from any cause.

### Statistical analysis

In accordance with NHANES analysis guidelines, we meticulously considered the intricate sampling designs and sample sizes during the data analysis process. Data were weighted to achieve a representative sample size for each sample. (Johnson et al., 2014). The weighting formula is as follows: 9 cycles of mobile examination center (MEC) weights = 4 years of MEC weights (1999–2002) × 2/9 + 2 years of MEC weights (2003–2016) × 1/9. All data analyses were conducted using the statistical software package R (http://www.r-project.org, version 4.2.3). First, the participants were divided into a training set and a validation set in the ratio of 7:3 (using the ‘caret’ package version 6.0–93, “createDataPartition” function). Continuous variables were presented as weighted means ± standard deviations. To compare differences between groups, Student’s t-test was employed. Meanwhile, categorical variables were expressed as frequencies and percentages and compared using Rao-Scott’s χ2 test. In addition, correlation analysis was performed using person correlation for continuous variables. Furthermore, variables in the training set underwent a filtering process through the LASSO regression. Variables that had non-zero coefficients in the LASSO regression model were chosen for building the nomogram prediction model. To confirm the statistical significance of the variables included in the nomogram model, Cox proportional hazard regression was employed. The accuracy of the risk prediction model was assessed through several metrics, including time-dependent receiver operating characteristic (ROC) curves, the concordance index (C-Index), calibration plots, and decision curve analysis (DCA) curves. These evaluations were conducted separately for the training and validation sets. The ROC curve area under the curve (AUC) has a value between 0.5 and 1. An AUC value close to 1 indicates the good performance of the prediction model (Harrell et al., 1982). The C-index is the proportion of the total number of pairs in which the predicted outcome agrees with the actual outcome in a two-by-two pairing of all samples. C-index >0.7 is considered to have good discriminatory power (Longato et al., 2020). The calibration plot is a scatter plot of the actual and predicted incidence, and if the curves are on the diagonal of the coordinates respectively indicates that the predictive power of the model is more accurate (Barda et al., 2020). The DCA curve avoids the problems of choosing the critical value of the ROC curve, sensitivity, and specificity, and directly calculates the net benefit in the clinical setting (Vickers et al., 2008). DCA curves that are above the two extreme values indicate that the model has good clinical applicability. In all the above analyses, a two-sided *p*-value less than 0.05 was considered statistically significant.

## Results

### Baseline characteristics of the study population categorized by training and validation sets

**FIGURE 1:**
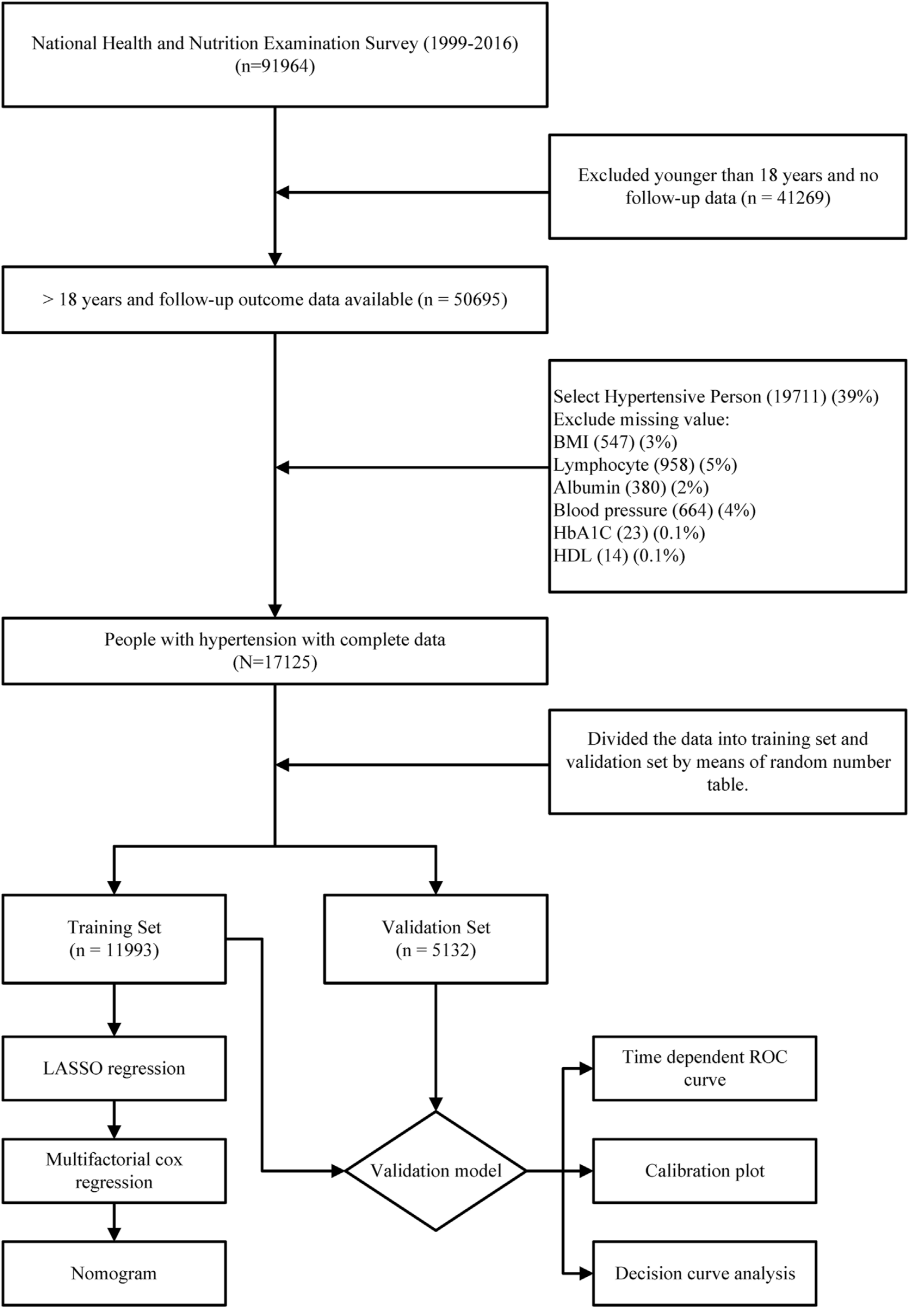
Flow chart of the entire process of inclusion criteria and statistical analysis of the hypertensive population in this study.

In this study, we initially included a total of 50,695 participants with valid follow-up data. Subsequently, after excluding individuals without hypertension (61% of the initial sample) and those with incomplete data, a final study population of 17,125 participants was established (Figure 1). The average age of the study participants was 56.5 years, with 8,480 (49.5%) being male and 8,645 (50.5%) being female. Over a median follow-up period of 109 months, 4,656 deaths occurred among the hypertensive population. For cross-validation purposes, the total number of cases was divided into a training set (11,993 participants) and a validation set (5,132 participants) in a 7:3 ratio. It is important to note that we applied weighting and adjustments to all results due to the complex sampling design utilized by NHANES. Within the training set, the mean age of participants was 56.5 years, consisting of 5,969 (49.4%) men and 6,024 (50.6%) women. During a median follow-up period of 108 months, 3,274 patients passed away. In contrast, the median age of participants in the validation set was 56.3 years, comprising 2,511 (49.0%) men and 2,621 (51.0%) women. Within this group, 1,382 patients experienced mortality during a median follow-up period of 110 months. When comparing the two groups, significant statistical differences were observed in terms of weight, BMI, monocyte counts, HbA1C levels, and the prevalence of Diatetes. There were no statistically significant variations in other general variables or laboratory test data. (Table 1).

**TABLE 1:** Baseline characteristics of training and validation sets.

	Overall (n = 17125)	Training set (n = 11993)	Validation set (n = 5132)	*p*-value
**Gender**				0.719
Male	8480 (49.5)	5969 (49.4)	2511 (49.0)	
Female	8645 (50.5)	6024 (50.6)	2621 (51.0)	
**Race**				0.300
Non-Hispanic white	4123 (24.1)	2865 (12.5)	1258 (13.3)	
Non-Hispanic black	2600 (15.2)	1766 (5.5)	834 (6.0)	
Mexican American	2339 (13.7)	1659 (9.7)	680 (9.9)	
Other race	8063 (47.1)	5703 (72.2)	2360 (70.9)	
**Marital**				0.249
With partner	9985 (58.3)	7010 (63.8)	2975 (62.1)	
Without partner	5125 (29.9)	3590 (25.3)	1535 (26.0)	
Never married	2015 (11.8)	1393 (10.9)	622 (11.9)	
**Education**				0.347
High school or above	14551 (85)	10223 (92.4)	4328 (92.0)	
Less than High School	2574 (15)	1770 (7.6)	804 (8.0)	
**Age, year**	56.45 ± 0.22	56.50 ± 0.25	56.31 ± 0.29	0.484
**Weight, kg**	86.99 ± 0.23	87.25 ± 0.26	86.36 ± 0.38	0.035
**Height, cm**	168.19 ± 0.12	168.23 ± 0.13	168.08 ± 0.22	0.543
**BMI, kg/m2**	30.63 ± 0.08	30.71 ± 0.09	30.42 ± 0.12	0.033
**WBC, 10^9/L**	7.40 ± 0.03	7.39 ± 0.04	7.42 ± 0.05	0.436
**Monocyte, 10^9/L**	0.58 ± 0.00	0.58 ± 0.00	0.59 ± 0.01	0.042
**Lymphocyte, 10^9/L**	2.12 ± 0.01	2.11 ± 0.01	2.15 ± 0.02	0.171
**Neutrophil, 10^9/L**	4.43 ± 0.02	4.44 ± 0.03	4.42 ± 0.03	0.660
**RBC, 10^12/L**	4.68 ± 0.01	4.68 ± 0.01	4.69 ± 0.01	0.651
**Hemoglobin, g/L**	14.29 ± 0.03	14.29 ± 0.03	14.29 ± 0.04	0.979
**PLT, 10^9/L**	253.22 ± 0.96	253.04 ± 1.11	253.63 ± 1.39	0.713
**HbA1c, %**	5.83 ± 0.01	5.84 ± 0.01	5.80 ± 0.01	0.041
**Albumin, g/L**	42.56 ± 0.05	42.52 ± 0.05	42.64 ± 0.07	0.114
**Cr, μmol/L**	83.06 ± 0.43	83.34 ± 0.52	82.37 ± 0.60	0.183
**UA, μmol/L**	343.52 ± 1.00	343.43 ± 1.15	343.73 ± 1.47	0.854
**Sodium, mmol/L**	139.11 ± 0.05	139.12 ± 0.05	139.09 ± 0.06	0.508
**Potassium, mmol/L**	4.02 ± 0.01	4.02 ± 0.01	4.01 ± 0.01	0.250
**HDL-C, mmol/L**	1.35 ± 0.01	1.35 ± 0.01	1.35 ± 0.01	0.454
**LDL-C, mmol/L**	3.04 ± 0.01	3.04 ± 0.01	3.03 ± 0.02	0.702
**TG, mmol/L**	1.72 ± 0.02	1.72 ± 0.02	1.71 ± 0.03	0.747
**SBP, mmHg**	134.25 ± 0.26	134.20 ± 0.28	134.35 ± 0.38	0.706
**DBP, mmHg**	74.09 ± 0.23	74.01 ± 0.24	74.28 ± 0.31	0.374
**Cardiovascular disease**				0.295
yes	3409 (19.9)	2372 (16.7)	1037 (17.5)	
no	13716 (80.1)	9621 (83.3)	4095 (82.5)	
**Diabetes**				0.002
yes	6423 (37.5)	4535 (33.6)	1888 (30.6)	
no	10702 (62.5)	7458 (66.4)	3244 (69.4)	
**Beta-blockers**				0.807
yes	4025 (23.5)	2817 (22.7)	1208 (22.5)	
no	13100 (76.5)	9176 (77.3)	3924 (77.5)	
**Calcium channel blockers**				0.741
yes	3421 (20)	2385 (17.0)	1036 (16.7)
no	13704 (80)	9608 (83.0)	4096 (83.3)	
**ACEI/ARB**				0.694
yes	6915 (40.4)	4820 (39.4)	2095 (39.8)	
no	10210 (59.6)	7173 (60.6)	3037 (60.2)	
**Diuretics**				0.036
yes	4949 (28.9)	3457 (27.0)	1492 (29.0)	
no	12176 (71.1)	8536 (73.0)	3640 (71.0)	
**Statins**				0.231
yes	4928 (28.8)	3466 (28.4)	1462 (27.2)	
no	12197 (71.2)	8527 (71.6)	3670 (72.8)	

Categorical data are presented as weighted means ± standard deviations, while continuous data are expressed as percentages.

Abbreviations: BMI, body mass index; WBC, white blood cell count; RBC, red blood cell count; Cr, Creatinine; UA, uric acid; TG: triglyceride; SBP, systolic blood pressure; DBP, diastolic blood pressure; LDL-C, Low-Density Lipoprotein Cholesterol; HDL-C, High-Density Lipoprotein Cholesterol; HbA1C, glycosylated hemoglobin; ACEI/ARB, Angiotensin-Converting Enzyme Inhibitors/Angiotensin II, receptor antagonists.

### Construction of clinical prediction models

**FIGURE 2:**
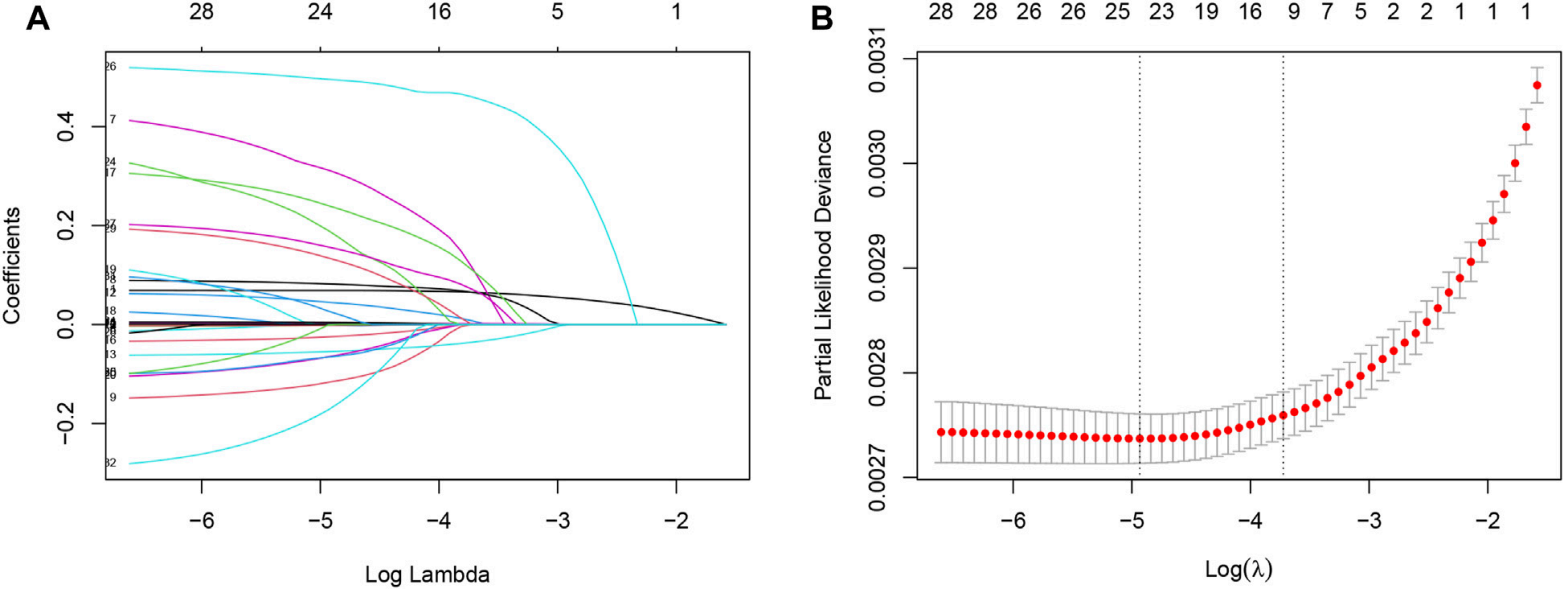
Least absolute shrinkage and selection operator (LASSO) regression models were used to select risk factors contributing to all-cause mortality in the American hypertensive population over a mean of 9 years. **(A)** LASSO coefficient curves for 32 Variables. X-axis is log (lambda) and Y-axis is partial regression coefficients. As log (lambda) increases, the compression parameter increases, and the absolute value of the biased regression coefficient decreases, possibly to zero, and is thus excluded. **(B)** The optimal parameter (lambda) in the LASSO model was selected using a five-fold cross-validation based on the minimum criterion. Partial likelihood deviation (binomial deviation) curves were plotted against log (lambda). Dashed vertical lines were drawn at the optimal values by using the minimum mean squared error and one standard deviation of the minimum mean squared error (1-SE criterion). The 9 hazard Variables corresponding to log (lambda)-1SE were used.

**FIGURE 3:**
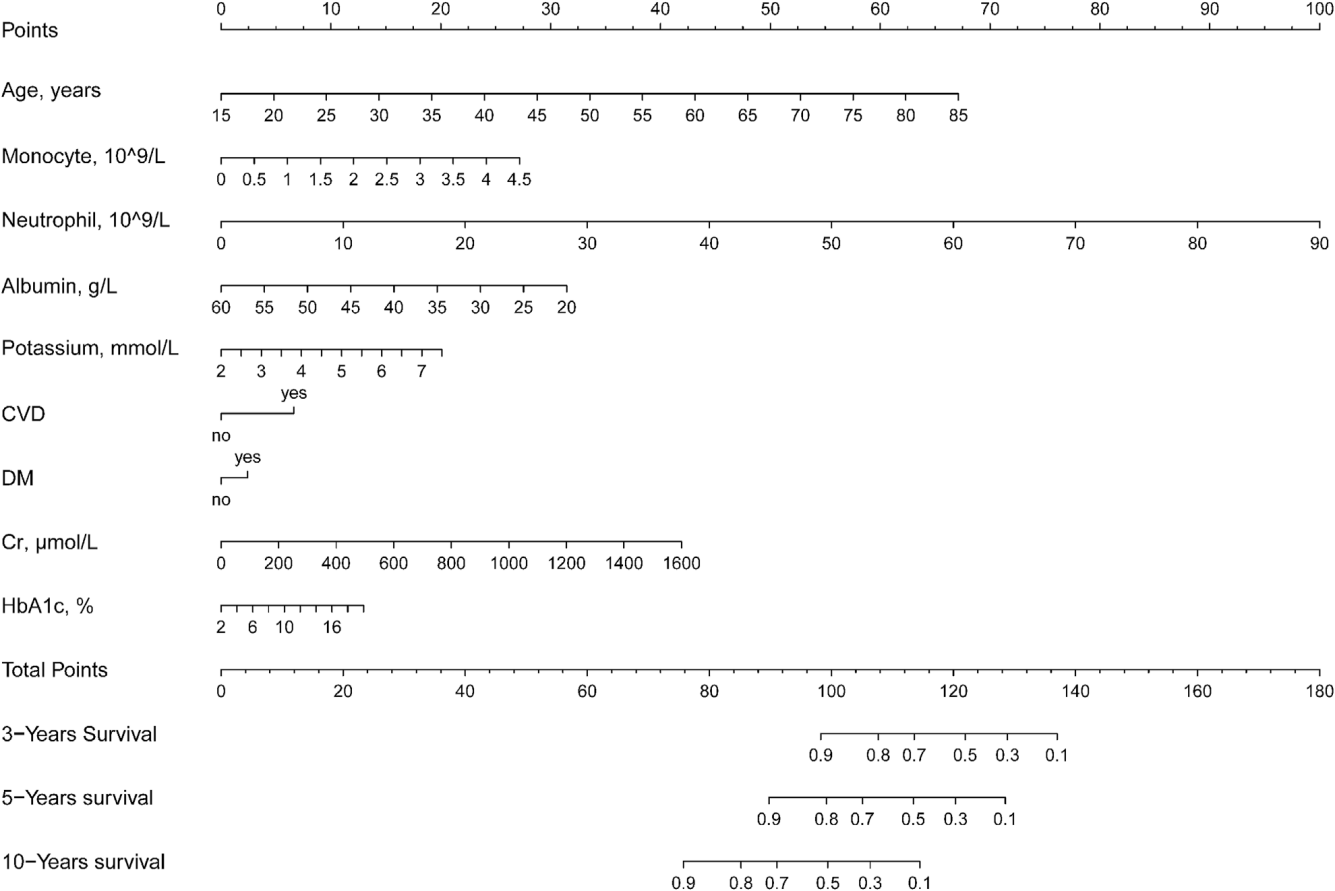
Nomogram to assess the risk of all-cause mortality in a hypertensive population over 3, 5, and 10 years.

To develop clinical prediction models, we employed LASSO regression to screen variables from an initial pool of 32 risk factors within the training set data (Figure 2A). Utilizing the regularization technique, we identified the optimal lambda value, which was situated within one standard deviation of the minimum mean square error of the minimum lambda (Figure 2B). This process resulted in the reduction of the potential predictor variables from 32 down to 9. The final variables of age, monocytes, neutrophils, serum albumin, serum potassium, history of CVD, history of diabetes, serum creatinine, and HbA1C were selected to participate in the construction of the nomogram (Figure 3). A multifactorial COX proportional hazard regression analyze was also applied to assess the validity of these variables (Table 2). To verify the representativeness of these characteristics, person correlation coefficients indicated that the above nine variables were correlated with other variables that were not included in the Nomogram (Supplementary Figure S1).

**TABLE 2:** Validation of each variable using multifactorial Cox regression based on coefficients and lambda.1se values from LASSO regression on the training set.

Characteristics	LASSO regression		Multifactorial COX regression analyze		
	Coefficients	λ.1se	Hazard ratio	95% CI	*p*-value
Age	0.064199506	0.024149	1.079	(1.074,1.084)	<0.001
Monocyte	0.079371971		1.619	(1.314,1.995)	<0.001
Neutrophil	0.061652841		1.092	(1.072,1.113)	<0.001
Albumin	−0.033333818		0.939	(0.926,0.953)	<0.001
Potassium	0.092171986		1.339	(1.193,1.503)	<0.001
Cardiovascular disease	0.453366023		1.696	(1.542,1.865)	<0.001
Diabetes	0.060332001		1.213	(1.089,1.351)	<0.001
Creatinine	0.001711841		1.002	(1.001,1.003)	<0.001
HbA1c	0.001873895		1.059	(1.001,1.120)	0.044

Coefficients: coefficients of each variable in LASSO, regression; Lambda.1se: lambda value of the simplest model within a variance of the mean value of the minimum target parameter.

### Method of using nomogram

The essence of nomogram is to visualize complex model formulations (Xie et al., 2021). In the present study, the formula for the Nomogram was as follows.

$$\begin{array}{c} \text{Score}=\left(0.99\times \text{Age}-14.4\right)+\left(\text{Monocyte}\times 0.60\right)+\left(\text{Neutrophil}\times 1.11\right)+\left(\text{Albumin}\times -0.78+47.2\right)\\ +\left(\text{Potassium}\times 3.65-7.31\right)+\left(\text{Cr}\times 0.026\right)+\left(\text{HbA}1C\times 0.72-1.44\right)+\left(\text{CVD}\times 6.62\right)+\left(\text{DM}\times 2.41\right) \end{array}$$

Therefore, to simplify the equation, we constructed the nomogram and showed how to use the method (Supplementary Figure S2). For example, if an adult with hypertension is 55 years old, has a monocyte count of 0.5 × 10^9/L, a lymphocyte count of 10 × 10^9/L, albumin of 40 g/L, a serum potassium concentration of 4 mmol/L, cardiovascular disease, no diabetes mellitus, a creatinine of 400 μmol/L, and an HbA1C of 5%. Then his total score was 95, with a survival rate of >90% at 3 years, about 85% at 5 years, and about 60% at 10 years.

### Validation of clinical prediction models

**FIGURE 4:**
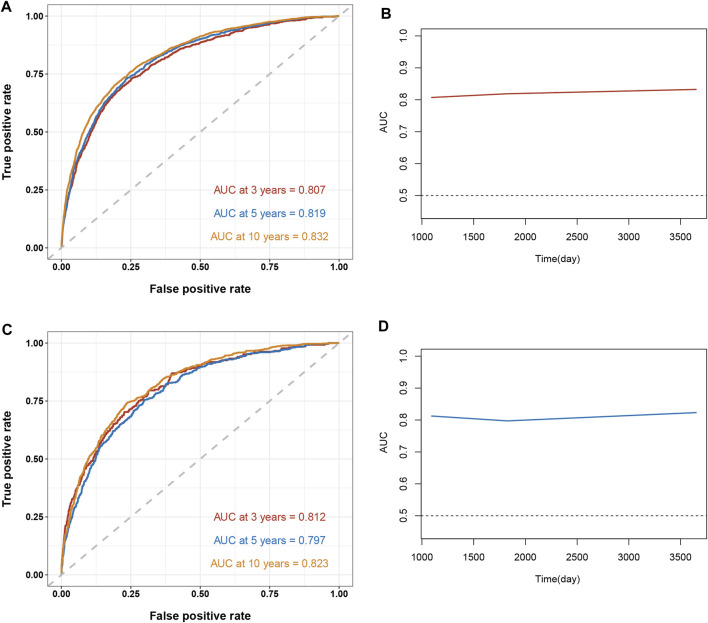
Time-dependent receiver operating curve (ROC) analysis and time-dependent AUC curves for the Nomogram model for predicting all-cause mortality in hypertensive patients. The ROC curve in red indicates a forecast cutoff of 3 years, blue indicates 5 years, and yellow indicates 10 years. **(A)** ROC curve for the training set. **(B)** AUC curve for the training set. **(C)** ROC curve for the validation set. **(D)** AUC curve for the validation set.

**FIGURE 5:**
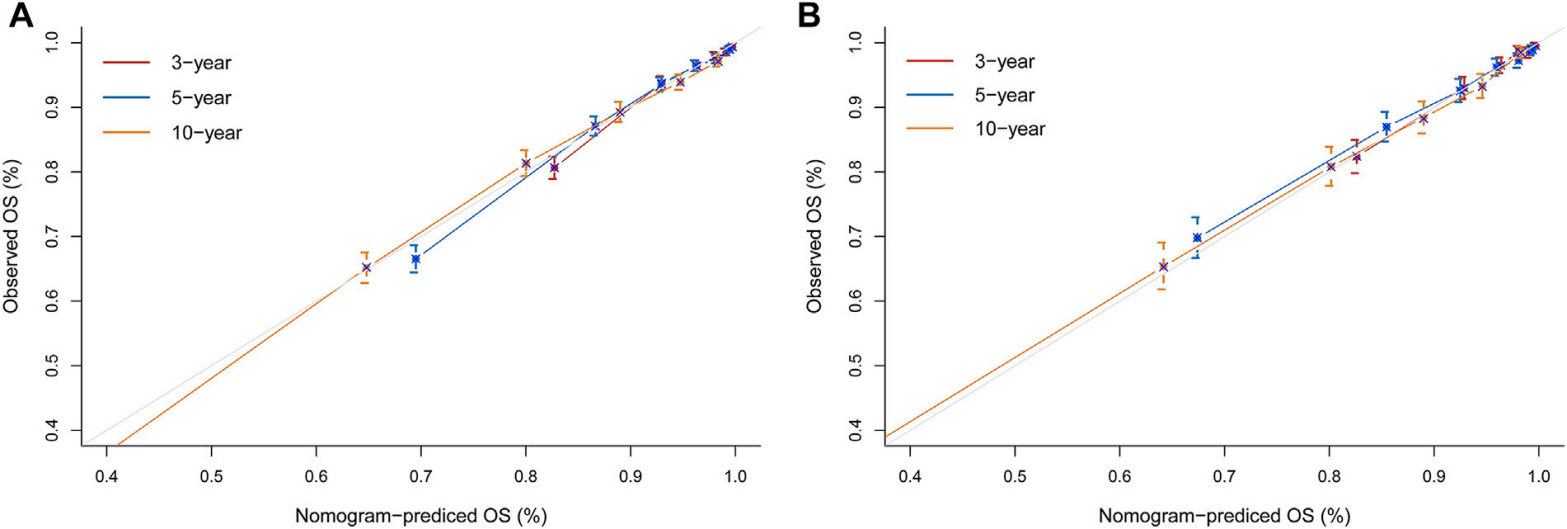
Nomogram calibration curves for predicting all-cause mortality risk in a hypertensive population at 3, 5, and 10 years. **(A)** Training set. **(B)** Validation set. X-axis represents the predicted risk of mortality. Y-axis represents the actual mortality. The red curve indicates a forecast time cutoff of 3 years, blue indicates 5 years, and yellow indicates 10 years. The dashed line on the diagonal represents the perfect prediction of the ideal model. The solid line represents the performance of the nomogram. A better fit to the dashed diagonal line represents a better prediction.

**FIGURE 6:**
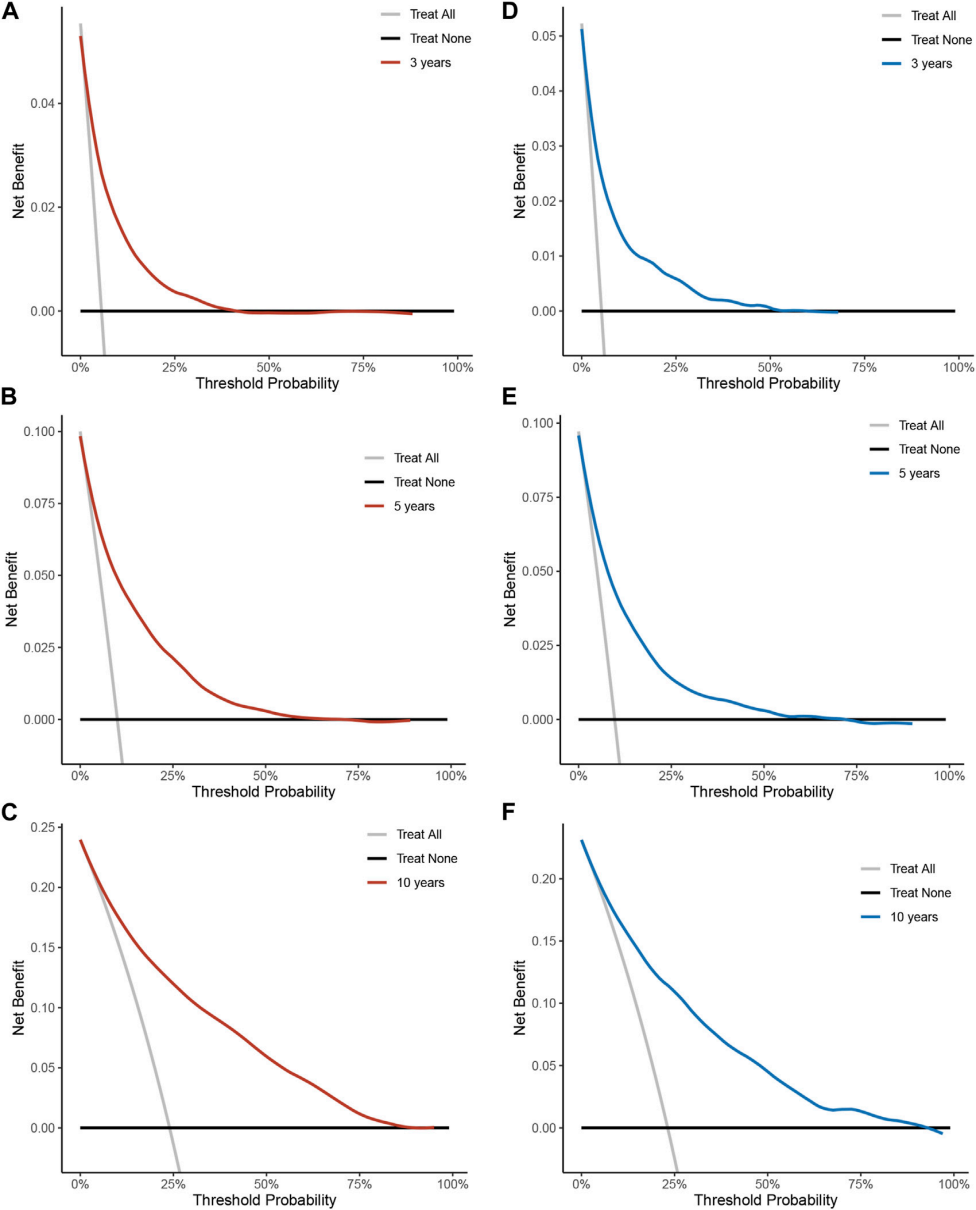
Decision curve analysis of all-cause mortality risk nomograms over 3, 5, 10 years in a hypertensive population. **(A)** Training set for 3 years. **(B)** Training set for 5 years. **(C)** Training set for 10 years. **(D)** Validation set for 3 years. **(E)** Validation set 5 years. **(F)** Validation set for 10 years. Y-axis represents net benefit. The dashed line represents the nomogram. The thick solid line represents the hypothesis of all mortality. The thin solid line represents the hypothesis of no disease mortality.

To assess the reliability of the established clinical prediction model, we conducted testing on both the training and validation datasets. First, we calculated the overall C-index of the model. In the training set, the C-index for the nomogram model was 0.800 (95% CI, 0.792–0.808; *p* < 0.001), while in the validation set, it was 0.793 (95% CI, 0.781–0.805; *p* < 0.001). Second, we employed a time-dependent receiver operating curve (ROC) analysis to evaluate the accuracy of the nomogram model in predicting the risk of all-cause mortality at 3, 5, and 10 years (Figure 4). The AUC values were 0.807, 0.819 and 0.833 for 3, 5 and 10 years in the training group, respectively. The AUC values were 0.812, 0.797 and 0.824 for 3, 5 and 10 years in the validation set, respectively. Finally, calibration curve validation (Figure 5) and DCA analysis (Figure 6) were performed on the data from the training and validation sets, respectively. The calibration curve demonstrates that the actual mortality (Y-axis) and the all-cause mortality (X-axis) fitted in the nomogram model are distributed around a straight line with a slope of 45° across the origin. The decision curve is in the horizontal coordinate range of 0.1–0.7 and lies above the two extreme lines of None and All, indicating that the model has good predictive power in this range.

## Discussion

In this research, we have devised and verified a straightforward clinical prediction model with a substantial and nationally representative dataset from NHANES. We employed machine learning techniques along with LASSO regression to accomplish this. We considered currently clinically accepted risk indicators for hypertension while selecting the most representative variables from a wide range of variables. Our ultimate findings reveal a significant association between the risk of all-cause mortality in hypertensive populations and the following variables: age, monocyte count, neutrophil count, serum albumin levels, serum potassium levels, cardiovascular history, history of diabetes, serum creatinine levels, and HbA1C levels. We developed a nomogram model with the above 9 variables. Through rigorous cross-validation, the nomogram model exhibited a reasonably strong ability to predict the risk of all-cause mortality within the hypertensive population.

Hypertension is positively associated with a range of serious life-threatening CVD such as angina pectoris, ischemic heart disease, heart failure, and myocardial infarction (Aune et al., 2021; Wu et al., 2023). Therefore, the prediction of mortality in hypertensive patients should receive more attention. To date, many models have been employed for predicting morbidity or mortality among individuals with hypertension. However, existing population-specific disease prediction models are not applicable to different regional populations due to various factors such as region, ethnicity, socioeconomic conditions, and disease prevention strategies. Pocock et al., 2001 developed a risk score system to prognosticate the risk of mortality in hypertensive patients using an equation. Martínez-Díaz et al., 2019 devised a scoring system to forecast 1-year all-cause mortality in hospitalized hypertensive individuals. Zhang et al., 2022 crafted a nomogram using NHANES data, but it targeted individuals aged over 65. To our knowledge, no predictive models currently exist to gauge the mortality risk specifically in the American adult hypertensive population. Hence, we have developed a nomogram capable of predicting all-cause mortality risk over 3, 5, and 10 years. This tool aims to enhance risk stratification among the adult hypertensive population in the United States.

In this prediction model, age is the most significant independent predictor. With aging, vascular endothelial cell function decreases, vascular elasticity vitality is gradually lost, and BP variability increases (Zixuan et al., 2020; Zhu M. et al., 2022). Especially in the hypertensive population, aging results in reversible loss of vascular function (Kruyer et al., 2015). In addition, comorbidities including diabetes mellitus, and CVD all exacerbate the risk in hypertensive patients. In alignment with prior research, the prevalence of diabetes at the time of hypertension diagnosis is linked to an elevated mortality risk. This association is largely attributable to the exacerbation of vascular endothelial injury due to oxidative stress induced by elevated glucose levels (Lip et al., 2016; Hubbard et al., 2019). When hypertension coexists with CVD, it further accelerates the progression of cardiovascular conditions. Consequently, this escalation leads to the development of a spectrum of severe complications, including arrhythmias, myocardial infarction and heart failure (Surendran et al., 2016).

In addition to the above traditional risk factors, monocytes, neutrophils, serum albumin, serum potassium, serum creatinine and HbA1C were also identified as the significant risk factors for all-cause mortality of hypertensive individuals. The inflammatory response plays a pivotal role in the onset and progression of hypertension, culminating in organ damage over time (Madhur et al., 2021). Circulating monocytes in hypertensive patients may further differentiate into macrophages. They then express tumor necrosis factor-α (TNF-α) and interleukin-1β (IL-1β) to promote immune response (Justin Rucker and Crowley, 2017). Moreover, neutrophils have been documented to involve in cardiovascular and renal damage in hypertension (McCarthy et al., 2021). During high BP, the autonomic nervous system can activate neutrophils, provoking vascular system damage, the release of reactive oxidants (ROS), and the promotion of endothelial dysfunction. Several clinical trials have demonstrated that monocytes and lymphocytes have an independent predictive effect on the prognosis of hypertensive patients (Tatsukawa et al., 2008; Liu et al., 2015; Ganjali et al., 2018). Serum albumin stands as a crucial marker reflecting the body’s nutritional status, with various functions including the maintenance of colloid osmotic pressure, preservation of microvascular integrity, antioxidant properties, and antithrombotic effects (Li et al., 2020). Several prior investigations have demonstrated that serum albumin can serve as a predictive factor for the prognosis of individuals with hypertension (Sun et al., 2017; Yılmaz et al., 2022). Potassium is a highly abundant cation in intracellular fluids and is directly involved in intracellular metabolic activities (Bischof et al., 2017). Concerns regarding the risk of hyperkalemia have garnered significant attention, particularly among those with chronic ailments. Research indicates that both low and high potassium levels are linked to all-cause mortality in hypertensive patients (Byrne et al., 2021). Therefore, it is particularly critical to maintain normal potassium levels. Serum creatinine (sCr) is a marker of kidney function (Allegretti et al., 2019). High blood creatinine is often associated with decreased glomerular filtration, causing water and sodium retention, which increases cardiac burden and the prevalence of CVD. A study conducted in China found an association between sCr levels and a 10 years cardiovascular risk in hypertensive patients (Chen et al., 2023). Glycated hemoglobin (HbA1c) is the binding product of hemoglobin to blood glucose and provides an estimation of long-term glycemic control (Hulme et al., 2017). Prolonged elevations in HbA1c can activate the advanced glycosylation end products (AGEs-RAGE) axis, subsequently leading to impaired endothelial function, release of vascular inflammatory factors, remodeling of vital arteries and atherosclerosis (Creager et al., 2003). One study has revealed an association between HbA1c levels and both cardiovascular mortality and all-cause mortality among the American population with hypertension (Chonchol et al., 2010).

### Strengths and limitations

This study possesses several notable strengths. Firstly, we drew a large, representative sample from NHANES, which was weighted in the data analysis, allowing our results to be generalized to the entire American population and providing a solid foundation for the findings. Secondly, we included predictors that are common in clinical practice. Patient demographic information or serum markers are routinely tested in hospitals, enhancing the utility of assessing prognosis. Thirdly, the model’s Robust predictive accuracy of this model assessed through cross-validation signifies the development of a more precise tool for gauging the risk of all-cause mortality among hypertensive patients. Nonetheless, certain limitations should be acknowledged. Firstly, the retrospective nature of this study necessitates prospective research to validate our results and ensure their generalizability. Secondly, the study’s population was confined to adult hypertensive patients in the United States, potentially limiting the model’s applicability to other countries and regions.

In conclusion, our study reveals that age, monocytes, neutrophils, serum albumin, serum potassium, cardiovascular disease, diabetes mellitus, serum creatinine, and HbA1C are all significant factors tied to the risk of all-cause mortality in individuals with hypertension. Leveraging these risk factors, we have crafted a nomogram that can effectively predict the risk of all-cause mortality in American adults afflicted with hypertension. This nomogram represents a valuable tool for clinicians, enabling them to assess prognosis and implement timely interventions for improved patient care.

## Data Availability

The datasets presented in this study can be found in online repositories. The names of the repository/repositories and accession number(s) can be found below: https://wwwn.cdc.gov/nchs/nhanes/search/default.aspx.
